# Determinants of Health Literacy and Its Associations With Health-Related Behaviors, Depression Among the Older People With and Without Suspected COVID-19 Symptoms: A Multi-Institutional Study

**DOI:** 10.3389/fpubh.2020.581746

**Published:** 2020-11-16

**Authors:** Binh N. Do, Phung-Anh Nguyen, Khue M. Pham, Hoang C. Nguyen, Minh H. Nguyen, Cuong Q. Tran, Thao T. P. Nguyen, Tien V. Tran, Linh V. Pham, Khanh V. Tran, Trang T. Duong, Thai H. Duong, Kien T. Nguyen, Thu T. M. Pham, Min-Huei Hsu, Tuyen Van Duong

**Affiliations:** ^1^Department of Infectious Diseases, Vietnam Military Medical University, Hanoi, Vietnam; ^2^Division of Military Science, Military Hospital 103, Hanoi, Vietnam; ^3^International Center for Health Information Technology, Taipei Medical University, Taipei, Taiwan; ^4^Department of Healthcare Information and Management, Ming Chuan University, Taoyuan City, Taiwan; ^5^Faculty of Public Health, Hai Phong University of Medicine and Pharmacy, Hai Phong, Vietnam; ^6^President Office, Hai Phong University of Medicine and Pharmacy, Hai Phong, Vietnam; ^7^Director Office, Thai Nguyen National Hospital, Thai Nguyen City, Vietnam; ^8^President Office, Thai Nguyen University of Medicine and Pharmacy, Thai Nguyen City, Vietnam; ^9^International Master/Ph.D. Program in Medicine, Taipei Medical University, Taipei, Taiwan; ^10^Department of Anesthesiology, Thu Duc District Hospital, Ho Chi Minh City, Vietnam; ^11^Director Office, Thu Duc District Health Center, Ho Chi Minh City, Vietnam; ^12^Health Management Training Institute, Hue University of Medicine and Pharmacy, Hue, Vietnam; ^13^Department of Health Economics, Corvinus University of Budapest, Budapest, Hungary; ^14^Director Office, Military Hospital 103, Hanoi, Vietnam; ^15^Department of Pulmonary and Cardiovascular Diseases, Hai Phong University of Medicine and Pharmacy Hospital, Hai Phong, Vietnam; ^16^Director Office, Hai Phong University of Medicine and Pharmacy Hospital, Hai Phong, Vietnam; ^17^Director Office, Hospital District 2, Ho Chi Minh City, Vietnam; ^18^Nursing Office, Tan Phu District Hospital, Ho Chi Minh City, Vietnam; ^19^Department of Internal Medicine, Thai Nguyen University of Medicine and Pharmacy, Thai Nguyen City, Vietnam; ^20^Department of Health Education, Faculty of Social Sciences, Behavior and Health Education, Hanoi University of Public Health, Hanoi, Vietnam; ^21^Graduate Institute of Data Science, Taipei Medical University, Taipei, Taiwan; ^22^School of Nutrition and Health Sciences, Taipei Medical University, Taipei, Taiwan

**Keywords:** COVID-19, older people, health literacy, health-related behaviors, depression, Vietnam

## Abstract

**Purpose:** We examined factors associated with health literacy among elders with and without suspected COVID-19 symptoms (S-COVID-19-S).

**Methods:** A cross-sectional study was conducted at outpatient departments of nine hospitals and health centers 14 February−2 March 2020. Self-administered questionnaires were used to assess patient characteristics, health literacy, clinical information, health-related behaviors, and depression. A sample of 928 participants aged 60–85 years were analyzed.

**Results:** The proportion of people with S-COVID-19-S and depression were 48.3 and 13.4%, respectively. The determinants of health literacy in groups with and without S-COVID-19-S were age, gender, education, ability to pay for medication, and social status. In people with S-COVID-19-S, one-score increment of health literacy was associated with 8% higher healthy eating likelihood (odds ratio, OR, 1.08; 95% confidence interval, 95%CI, 1.04, 1.13; *p* < 0.001), 4% higher physical activity likelihood (OR, 1.04; 95%CI, 1.01, 1.08, *p* = 0.023), and 9% lower depression likelihood (OR, 0.90; 95%CI, 0.87, 0.94; *p* < 0.001). These associations were not found in people without S-COVID-19-S.

**Conclusions:** The older people with higher health literacy were less likely to have depression and had healthier behaviors in the group with S-COVD-19-S. Potential health literacy interventions are suggested to promote healthy behaviors and improve mental health outcomes to lessen the pandemic's damage in this age group.

## Introduction

The COVID-19 pandemic has upended public health systems around the globe (1, 2), and spurring millions of health, research and administrative professionals to seek ways to mitigate transmission and mortality (3, 4). Older people are at high risk of more severe health conditions from COVID-19 disease (5, 6). By the time the virus killed 350,000 people, the over-60 death rate was estimated at 75%. The pandemic also has caused panic and mental illness, especially for the elderly (7, 8). Quarantine and lockdown contain infection but they negatively impact mental health (9–11). Different approaches are needed to mitigate the pandemic's psychological effects (12, 13).

Health literacy (HL) is known as a crucial means to appraise health-related information for preventing non-communicable and infectious diseases. It helps people achieve better quality care and improves disease management, lifestyle, and health outcomes (14, 15). Health literacy is considered a crucial element in public health strategies to protect people from disease (16, 17). This has never been more important than during the COVID-19 epidemic (18, 19). Vietnam has a high risk of coronavirus infection, having a long border with China, and Vietnamese people have lower health literacy scores when ranked among other Asian countries (20). Finding factors associated with health literacy can assist in planning interventions to reduce health inequalities during the epidemic.

This study explores determinants of health literacy and its associations with health-related behaviors and depression among older people with and without suspected COVID-19 symptoms (S-COVID-19-S).

## Materials and Methods

### Study Design

We conducted a cross-sectional study on outpatient department (OPD) visitors 14 February−2 March 2020. The study was reviewed and approved by the nine participating hospitals and health centers as well as the Institutional Ethical Review Committee of Hanoi School of Public Health in Vietnam (IRB No. 029/2020/YTCC-HD3).

### Study Participants and Settings

Patients recruited in the study were ages 60–85 years, able to communicate in Vietnamese, and visited an OPD during the study period. Participants were excluded if they were in any emergency condition or if they were diagnosed with psychotic disorders, dementia or blindness. The process of recruiting participants is detailed in a previous study (15).

We included 928 patients aged 60–85 years in the analysis, including 152 from Thai Nguyen National Hospital in Thai Nguyen Province, 56 from Military Hospital 103 in Hanoi; 162 from Hai Phong University of Medicine, and Pharmacy Hospital, 281 from Kien Thuy District Health Center, in Hai Phong city; 141 from Trieu Phong District Health Center in Quang Tri province; 40 from Thu Duc Hospital, 27 from Tan Phu District Hospital, 23 from Hospital District 2, and 46 from Thu Duc District Health Center, in Ho Chi Minh city.

### Data Collection Procedure

The interviewers (e.g., nurses, staff, and medical students) at each hospital or health center had received 4 h of training for the data collection; the sessions were led by two senior researchers with a detailed protocol. Technical guidance for prevention and control of COVID-19 disease was also provided during the training, including mask use, hand washing and physical distancing (4).

Interviewers invited OPD visitors to participate in the survey after signing consent form. The interviews were conducted at the OPDs using printed questionnaires that took about 20 min to complete. Personal information (e.g., name, identification) was anonymized before the analysis.

### Measurements

#### Demographic Characteristics and Clinical Indicators

Socio-demographic indicators assessed included age (date of birth), gender (female, male), marital status (never, ever married), education (illiterate or elementary school, junior high school, high school, college/university, or above), occupation (employed, business owner, others), social status (low, middle, or high level), and ability to pay for medication (very difficult to very easy).

Participants visiting the OPD were asked why they sought healthcare services, and were screened for suspected COVID-19 symptoms (S-COVID-19-S). Patients were classified in the S-COVID-19-S group if they carried any of the common symptoms (e.g., fever, cough, dyspnea, fatigue, myalgia, anorexia, or sore throat) or uncommon symptoms (e.g., confusion, headache, rhinorrhea, hemoptysis, chest pain, conjunctivitis, bronchial breath sounds, diarrhea, cyanosis, and nausea/vomiting) (21). In addition, height (cm), weight (kg), body mass index (BMI, kg/m^2^), and comorbid conditions (Charlson comorbidity index diseases) were assessed.

#### Health-Related Behaviors

Health-related behaviors included current smoking status (no vs. yes), drinking status (no vs. yes), and eating behaviors during the COVID-19 outbreak (unchanged or less healthy, or healthier). The seven-item International Physical Activity Questionnaire short version (IPAQ-SF) asked patients' activities (vigorous, moderate, walking, and sitting) over the past 7 days before the OPD visiting date (22, 23). The overall physical activity score as MET-min/week was calculated for each subject and used in the analysis (24).

#### Health Literacy

We used a short-form questionnaire (HLS-SF12) to measure health literacy (15, 25, 26). People were asked about the perceived difficulty of 12 items on a 4-point Likert scale (1 = very difficult to 4 = very easy). We calculated the general health literacy (HL) index score using the formula (1):

(1) $$\begin{array}{l} HL\;index=\left(M-1\right)*50/3 \end{array}$$

where the *HL index* is ranged from 0 to 50; *M* is the mean of 12 items of HLS-SF12. The higher HL index indicates a greater HL level (25, 27).

#### Depression

We assessed depression using patient health questionnaire with 9 items (PHQ-9) that had been validated and used in Vietnam (28–30). Patients rated each item using the 4-point Likert scale from 0 = not at all to 3 = almost every day for the past 14 days. The depression scores range from 0 to 27, with those scoring ≥10 classified as having depression (31).

### Statistical Analysis

The distributions of studied variables were explored using descriptive analysis. The Student's *t*-test and Chi-square tests were used appropriately for continuous and categorical variables. The determinants of health literacy were examined using simple and multiple linear regression analysis. Next, the simple and multiple binary logistic regression analyses were used to examine the associations of health literacy (as a predictor/independent variable) with binary outcome variables such as BMI (normal weight vs. overweight/obese), smoking status (non-smoking vs. smoking), drinking (non-drinking vs. drinking), eating behavior (eat less healthily or unchanged vs. eating healthier diet), depression (not depressed vs. depressed). The simple and multiple multinomial logistic regression analyses were used to examine the association between health literacy and physical activity (tertile-1 vs. tertile-2, tertile-3). Variables showing significant associations with outcome variables in simple regression models were selected for multiple regression models. In order to avoid multicollinearity, the Spearman's correlation coefficient test was used to check associations between independent variables. If independent variables correlated with one another at *rho* ≥ 0.3, one representative independent variable was selected to the multiple regression model. The significance level was set at a *p*-value < 0.05. Data were analyzed using SPSS for windows, version 20.0 (IBM Corp, Armonk, NY, USA).

## Results

### Demographic Characteristics of Participants

Out of sample, percentages of older people with S-COVID-19-S, and depression were 48.3% (448/928), and 13.4% (124/928), respectively. The mean age and health literacy scores were 68.2 ± 6.51, and 25.7 ± 8.09, respectively. The proportion of people with S-COVID-19-S varied with different categories of educational attainment, occupation, comorbidity, ability to pay for medication, social status, BMI, drinking, physical activity, and depression. People with S-COVID-19-S also had lower HL score than those without (Table 1).

**Table 1:** Characteristics of patients with and without suspected COVID-19 symptoms.

**Variables**	**Total (*n* = 928)**	**Without S-COVID-19-S^**a**^ (*n* = 480)**	**With S-COVID-19-S^**a**^ (*n* = 448)**	
	***n* (%)**	***n* (%)**	***n* (%)**	***p*-value^**b**^**
Age, year				0.066
60–70	648 (69.8)	348 (72.5)	300 (67.0)	
71–85	280 (30.2)	132 (27.5)	148 (33.0)	
Gender				0.969
Women	522 (56.3)	270 (56.3)	252 (56.3)	
Men	405 (46.6)	210 (43.8)	195 (43.5)	
Marital status				0.742
Never married	33 (3.6)	18 (3.8)	15 (3.3)	
Ever married	895 (96.4)	462 (96.3)	433 (96.7)	
Education				0.019
Elementary school or illiterate	228 (24.6)	121 (25.2)	107 (23.9)	
Junior high school	293 (31.6)	166 (34.6)	127 (28.3)	
High school	199 (21.4)	102 (21.3)	97 (21.7)	
College/university or above	206 (22.2)	89 (18.5)	117 (26.1)	
Occupation				<0.001
Employed	34 (3.7)	19 (4.0)	15 (3.3)	
Business owner	282 (30.4)	102 (21.3)	180 (40.2)	
Others	609 (65.6)	358 (74.6)	251 (56.0)	
Comorbidity				<0.001
None	602 (64.9)	278 (57.9)	324 (72.3)	
One or more	323 (34.8)	196 (40.8)	124 (27.7)	
Ability to pay for medication				<0.001
Very or fairly difficult	544 (58.6)	217 (45.2)	327 (73.0)	
Very or fairly easy	383 (41.3)	262 (54.6)	121 (27.0)	
Social status				<0.001
Low	197 (21.2)	77 (16.0)	120 (26.8)	
Middle or high	730 (78.7)	402 (83.8)	328 (73.2)	
BMI, kg/m^2^				<0.001
Normal weight (BMI <25.0)	800 (86.2)	395 (82.3)	405 (90.4)	
Overweight/obese (BMI ≥ 25.0)	126 (13.6)	84 (17.5)	42 (9.4)	
Current smoking status				0.384
Not smoking	819 (88.3)	427 (89)	392 (87.5)	
Smoking	105 (11.3)	50 (10.4)	55 (12.3)	
Current drinking status				0.390
Not drinking	711 (76.6)	364 (75.8)	347 (77.5)	
Drinking	207 (22.3)	113 (23.5)	94 (21.0)	
Eating behavior^c^				0.030
Less healthy diet or unchanged	754 (81.3)	404 (84.2)	350 (78.1)	
Healthier diet	165 (17.8)	73 (15.2)	92 (20.5)	
Physical activity, MET-min/wk				<0.001
Tertile-1 (MET-min/wk <500)	397 (42.8)	178 (37.1)	219 (48.9)	
Tertile-2 (500 ≤ MET-min/wk <1,000)	141 (15.2)	54 (11.3)	87 (19.4)	
Tertile-3 (MET-min/wk ≥ 1,000)	390 (42.0)	248 (51.7)	142 (31.7)	
HL index				
Mean ± SD	25.7 ± 8.09	26.7 ± 8.32	24.5 ± 7.70	<0.001
PHQ				
Not depressed (PHQ <10)	804 (86.6)	457 (95.2)	347 (77.5)	<0.001
Depressed (PHQ ≥ 10)	124 (13.4)	23 (4.8)	101 (22.5)	

S-COVID-19-S, suspected COVID-19 symptoms; BMI, body mass index; HL index, health literacy index; PHQ, patient health questionnaire score; MET-min/wk, metabolic equivalent task-minutes for a week.

a With and without S-COVID-19-S groups indicate patients who had suspected COVID-19 symptoms and those who did not, respectively.

b The p-value is computed by Student t-test with continuous variables and Chi-square or Fisher exact test with category variables.

c *People were asked whether their eating behavior was worse, better, or unchanged during COVID-19 outbreak as compared to before the outbreak*.

### Determinants of Health Literacy

Table 2 illustrates the determinants of health literacy for both groups with and without S-COVID-19-S. We checked correlations among the independent variables and found education and social status moderately correlated for people without S-COVID-19-S (rho = 0.36; Supplementary Table 1). Social status was selected into the multiple linear regression model. There was no moderate or high correlation among confounders for people with S-COVID-19-S (Supplementary Table 2). Therefore, all independent variables in the simple regression model were retained in the multiple linear regression model. The results of multiple linear regression analysis show that in comparison to the 60–70 years group, people 71–85 had lower health literacy (regression coefficient, B, −4.36; 95% confidence interval, 95% CI, −5.95, −2.77; *p* = 0.001, for participants with S-COVID-19-S; and B, −3.74; 95% CI, −5.05, −2.43; *p* < 0.001, for those without S-COVID-19-S). Men had higher health literacy scores than women (B, 1.77; 95% CI, 0.42, 3.13; *p* = 0.01 for the S-COVID-19-S group; and B, 1.94; 95% CI, 0.42, 3.46; *p* = 0.013, for the without S-COVID-19-S group). People with higher educational attainment had higher health literacy scores (B, 3.28 ~ 4.59, *p* < 0.001, for the group with S-COVID-19-S). People with a better ability to pay for medication had higher health literacy scores than their counterparts (B, 5.69; 95% CI, 4.33, 7.06; *p* < 0.001, for the group with S-COVID-19-S; and B, 1.53; 95% CI, 0.16, 2.90; *p* = 0.028). Finally, people with higher social status had higher health literacy scores (B, 3.33, 95%CI, 1.327, 5.33, *p* = 0.001, for the group without S-COVID-19-S; and B, 1.65 (0.22, 3.08), *p* = 0.024, for the group with S-COVID-19-S; Table 2).

**Table 2:** Factors associated with health literacy among participants with and without suspected COVID-19 symptoms.

**Variables**	**Without S-COVID-19-S**^**a**^				**With S-COVID-19-S**^**a**^			
	**Unadjusted B-Coef (95% CI)^**b**^**	***p*-value**	**Adjusted B-Coef (95% CI)^**c**^**	***p*-value**	**Unadjusted B-Coef (95% CI)^**b**^**	***p*-value**	**Adjusted B-Coef (95% CI)^**c**^**	***p*-value**
Age, years								
60–70	0.00		0.00		0.00		0.00	
71–85	−5.88 (−7.50, −4.27)	<0.001	−4.36 (−5.95, −2.77)	0.001	−5.66 (−7.09, −4.22)	<0.001	−3.74 (−5.05, −2.43)	<0.001
Gender								
Women	0.00		0.00		0.00		0.00	
Men	2.40 (0.89, 3.91)	0.002	1.94 (0.42, 3.46)	0.013	2.48 (1.05, 3.91)	0.001	1.77 (0.42, 3.13)	0.010
Marital status								
Never married	0.00		0.00		0.00		0.00	
Ever married	5.30 (1.31, 9.29)	0.009	3.11 (−0.52, 6.74)	0.093	−4.73 (−8.63, −0.84)	0.017	−1.93 (−5.07, 1.22)	0.230
Education								
Elementary school or illiterate	0.00				0.00		0.00	
Junior high school	7.56 (5.85, 9.28)	<0.001			4.26 (2.41, 6.11)	<0.001	3.28 (1.65, 4.90)	<0.001
High school	9.84 (7.91, 11.76)	<0.001			7.16 (5.20, 9.12)	<0.001	4.24 (2.47, 6.03)	<0.001
College/university or above	10.90 (8.87, 12.94)	<0.001			7.35 (5.47, 9.23)	<0.001	4.59 (2.88, 6.30)	<0.001
Occupation								
Employed	0.00		0.00		0.00		0.00	
Business owner	−3.37 (−7.54, 0.79)	0.112	−2.96 (−6.72, 0.80)	0.123	−4.36 (−8.31, −0.40)	0.031	−0.28 (−3.61, 3.05)	0.870
Others	−3.19 (−7.11, 0.74)	0.111	−1.67 (−5.20, 1.86)	0.352	−6.28 (−10.18, −2.37)	0.002	−1.26 (−4.53, 2.00)	0.448
Comorbidity								
None	0.00		0.00		0.00		0.00	
One or more	−1.71 (−3.24, −0.18)	0.029	−1.06 (−2.42, 0.31)	0.129	−2.59 (−4.18, −1.01)	0.001	−0.82 (−2.14, 0.50)	0.222
Ability to pay for medication								
Very or fairly difficult	0.00		0.00		0.00		0.00	
Very or fairly easy	2.58 (1.08, 4.08)	0.001	1.53 (0.16, 2.90)	0.028	6.21 (4.69, 7.72)	<0.001	5.69 (4.33, 7.06)	<0.001
Social status								
Low	0.00		0.00		0.00		0.00	
Middle or high	8.39 (6.47, 10.32)	<0.001	3.33 (1.327, 5.33)	0.001	4.43 (2.86, 6.01)	<0.001	1.65 (0.22, 3.08)	0.024

B-Coef, regression coefficient; CI, confidence interval.

a With and without S-COVID-19-S groups indicate patients with suspected COVID-19 symptoms and those without.

b The simple linear regression model was used.

c *The multiple linear regression model was used*.

### Health Literacy and Its Consequences

Table 3 presents associations of health literacy (HL) and related outcomes (e.g., BMI, smoking status, drinking status, eating behavior, physical activity, and depression). To adjust for potential confounders of the association between HL and consequences, we conducted the analyses to explore the potential determinants of each outcome. The results are shown in Supplementary Tables 3–8. The results of multiple logistic regression analysis show that people with higher health literacy scores ate healthier diets in the group with S-COVID-19-S (odds ratio, OR, 1.08; 95% confidence interval, 95% CI, 1.04, 1.13; *p* < 0.001). Similar results were found with those who reported more physical activity in the S-COVID-19-S group (OR, 1.04; 95% CI, 1.01, 1.08; *p* = 0.023 for tertile-3). People with higher HL scores had a lower likelihood of depression (OR, 0.91; 95% CI, 0.87, 0.94; *p* < 0.001; Table 3).

**Table 3:** Health literacy as a predictor associates with body mass index, health-related behaviors, and depression among participants with and without suspected COVID-19 symptoms.

**Health literacy (1-score increment)**	**Overweight/obese (BMI** **≥** **25.0 kg/m**^**2**^**)**^**a**^		**Smoking**^**a**^		**Drinking**^**a**^		**Healthier diet**^**a**^		**Physical activity (Tertile-2)**^**a**^		**Physical activity (Tertile-3)**^**a**^		**Depression (PHQ** **≥** **10)**^**a**^	
	**OR (95% CI)**	***p***	**OR (95% CI)**	***p***	**OR (95% CI)**	***p***	**OR (95% CI)**	***p***	**OR (95% CI)**	***p***	**OR (95% CI)**	***p***	**OR (95% CI)**	***p***
Without S-COVID-19-S														
Model 1	1.02 (0.99, 1.05)	0.226	1.02 (0.98, 1.06)	0.287	1.02 (1.00, 1.05)	0.108	1.07 (1.03, 1.11)	<0.001	1.00 (0.97, 1.04)	0.870	1.03 (1.00, 1.05)	0.032	0.96 (0.92, 1.00)	0.066
Model 2	1.01 (0.98, 1.04)	0.590	1.00 (0.96, 1.04)	0.975	0.99 (0.95, 1.02)	0.390	1.04 (0.99, 1.08)	0.103	0.99 (0.95, 1.03)	0.639	0.99 (0.97, 1.02)	0.721	1.02 (0.96, 1.09)	0.461
With S-COVID-19-S														
Model 1	1.03 (0.99, 1.08)	0.191	1.01 (0.98, 1.05)	0.474	1.03 (1.00, 1.07)	0.044	1.11 (1.07, 1.15)	<0.001	1.02 (0.99, 1.05)	0.255	1.08 (1.04, 1.11)	<0.001	0.91 (0.88, 0.94)	<0.001
Model 2	1.01 (0.96, 1.06)	0.806	1.00 (0.96, 1.04)	0.948	0.98 (0.94, 1.03)	0.378	1.08 (1.04, 1.13)	<0.001	1.00 (0.96, 1.04)	0.952	1.04 (1.01, 1.08)	0.023	0.91 (0.87, 0.94)	<0.001

OR, odd ratio; CI, confidence interval; BMI, body mass index; PHQ, patient health questionnaire; S-COVID-19-S, suspected COVID-19 symptoms.

a Reference groups were normal weight (BMI <25.0 kg/m^2^), not smoking, not drinking, less healthy diet or unchanged, physical activity (Tertile-1), non-depression, respectively. Model 1: The unadjusted logistic regression model. Model 2: The adjusted logistic regression model. For BMI, the model was adjusted for education (for with S-COVID-19-S group only), occupation (for with S-COVID-19-S group only), and social status (for both groups) (Supplementary Table 3), full model is presented in Supplementary Table 9. For smoking status, the model was adjusted for gender (for both groups) (Supplementary Table 4), full model is presented in Supplementary Table 10. For drinking status, the model was adjusted for age (for without S-COVID-19-S group only), gender (for both groups), education (for both groups), occupation (for without S-COVID-19-S group only), and social status (for with S-COVID-19-S group only) (Supplementary Table 5), full model is presented in Supplementary Table 11. For eating behavior, the model was adjusted for age (for with S-COVID-19-S group only), gender (for with S-COVID-19-S group only), education (for both groups), occupation (for both groups), comorbidity (for with S-COVID-19-S group only), and social status (for both groups) (Supplementary Table 6), full model is presented in Supplementary Table 12. For physical activity, the model was adjusted for age (for both groups), education (for both groups), occupation (for with S-COVID-19-S group only), comorbidity (for with S-COVID-19-S group only), ability to pay for medication (for without S-COVID-19-S group only), and social status (for with S-COVID-19-S group only) (Supplementary Table 7), full model is presented in Supplementary Table 13. *For depression, the model was adjusted for age (for both groups), marital status (for without S-COVID-19-S group only), education (for both groups), and social status (for both groups) (Supplementary Table 8), full model is presented in Supplementary Table 14*.

## Discussion

Our study shows men had higher health literacy scores compared to women for both with and without S-COVID-19-S groups. Previous studies showed men facing higher risks of worse health outcomes and death from COVID-19 disease, especially among older adults (32–35). Similarly, older people (ages 71–85 years) had lower health literacy compared to the younger group (ages 60–70 years) in both with and without S-COVID-19-S groups. The findings were consistent with other studies finding health literacy levels lower among elders in various nations and periods (36, 37). Likewise, higher levels of education and social status were associated with higher health literacy scores in older people, which is in line with previous studies (38, 39). Therefore, improving health literacy might be a strategic approach to prevent COVID-19 and minimize its consequences, especially in men and the older people. In addition, active engagement of the elderly is encouraged to contain the pandemic (40, 41). Governments must provide detailed, timely and accurate information regarding the epidemic, particularly about prevention efforts and self-protective behaviors that minimize new infections (42–44). Vietnam's Ministry of Health has led all health institutions and related sectors to collaborate with the public against the COVID-19 epidemic (45). The government has encouraged people to enhance behaviors such as washing hands, wearing masks, and following updated health-related information to prevent the disease and improve health literacy (46).

The COVID-19 pandemic has devastated economies and labor markets, especially reducing jobs and workers' earnings (47). Vietnam's GDP is $2,740, lower than many industrializing nations, so its people particularly fear the pandemics' impacts on household income, such as not able to cover daily living costs or health care expenses (48). Our study shows elders with better ability to pay medication had higher health literacy scores in both with and without S-COVID-19-S groups. This evidence calls for a quick response from governments in terms of stimulus packages to cover food, water, essential goods, basic health services, and medical costs during the crisis (46).

In the current study, we found health literacy significantly associated with healthier diet and physical activity only in older people with S-COVID-19-S. This can be explained by those participants facing higher projected risks of coronavirus infection and severe outcomes. They arguably have the most to gain from practicing healthy lifestyles (e.g., healthy dietary intake and more physical activity) to protect and improve their health-related quality of life (46, 49).

One important finding was that higher health literacy scores were associated with lower likelihood of depression in older people with S-COVID-19-S. This finding is similar to previous studies (50, 51). In our previous study, higher health literacy scores also were associated with lower fear of COVID-19 and lower likelihood of depression (15). We observed that nearly 13% of elders had depressive symptoms with 22.5 and 4.8% of participants with and without S-COVID-19-S, respectively. This might indicate that the uncertain progression of the COVID-19 epidemic affects mental health possibly leading to hypochondriasis, worry about being infected, and fear of the uncontrollable epidemic's consequences (52).

The study has several limitations. First, causality cannot be generated on the basis of a cross-sectional design. The findings could be considered for further studies regarding the pandemic, especially in elderly participants. Second, the study was conducted during the sensitive time period of the global COVID-19 pandemic, when all participants and interviewers might have been at risk of infection. Researchers and leaders of hospitals and health centers made great efforts to protect the safety of study participants, and fortunately there were no new cases during the data collection period. In addition, have selected 9 hospitals and health centers in three parts of Vietnam, yet the sample may not fully-represent the general Vietnamese population. Finally, while we cannot follow-up with the participants to assess long-term associations, future longitudinal studies with larger samples are suggested to confirm these findings.

## Conclusions

In groups with and without S-COVID-19-S, the factors of age, gender, education, ability to pay for medication, and social status were significantly associated with health literacy. Elders with higher health literacy had greater likelihood of healthier behavior (e.g., healthy eating, physical exercise) and lower likelihood of depression, especially in the S-COVID-19-S group. Because improved health literacy protects elders, our findings should be helpful for policy-makers worldwide.

## Data Availability Statement

The raw data supporting the conclusions of this article will be made available on reasonable request to the corresponding author.

## Ethics Statement

The study was reviewed and approved the Institutional Ethical Review Committee of Hanoi University of Public Health, Vietnam (IRB No. 029/2020/YTCC-HD3).

## Author Contributions

BD, P-AN, and TD analyzed the data and drafted the manuscript. BD, P-AN, KP, HN, MN, CT, TN, TT, LP, KT, TTD, THD, KN, TP, M-HH, and TD contributed to conceptualization, investigation, methodology, validation, and writing review and editing. BD, P-AN, KP, HN, MN, CT, TN, TT, LP, KT, TTD, THD, KN, TP, and TD conducted data curation.

## Conflict of Interest

The authors declare that the research was conducted in the absence of any commercial or financial relationships that could be construed as a potential conflict of interest.
