# Supplementary material for: Determinants of Health Literacy and Its Associations With Health-Related Behaviors, Depression Among the Older People With and Without Suspected COVID-19 Symptoms: A Multi-Institutional Study
Source: Front Public Health. 2020 Nov 16;8:581746. doi: 10.3389/fpubh.2020.581746 (PMC7703185; doi:10.3389/fpubh.2020.581746)
Supplement: Supplementary file 1 [file Data_Sheet_1.docx]

**Determinants of health literacy and its associations with health-related behaviors, depression among the older people with and without suspected COVID-19 symptoms: A multi-institutional study**

**Appendix**

[SUPPLEMEENTARY TABLE 1 | Spearman's correlation coefficient of study variables for people without S-COVID-19-S. 3](#_Toc50492267)

[SUPPLEMEENTARY TABLE 2 | Spearman's correlation coefficient of study variables for people with S-COVID-19-S. 4](#_Toc50492268)

[SUPPLEMEENTARY TABLE 3 | Factors associated overweight/obesity among people with and without S-COVID-19-S. 5](#_Toc50492269)

[SUPPLEMEENTARY TABLE 4 | Factors associated with smoking behavior among people with and without S-COVID-19-S. 6](#_Toc50492270)

[SUPPLEMEENTARY TABLE 5 | Factors associated with drinking behavior among people with and without S-COVID-19-S. 7](#_Toc50492271)

[SUPPLEMEENTARY TABLE 6 | Factors associated with eating behavior among people with and without S-COVID-19-S. 8](#_Toc50492272)

[SUPPLEMEENTARY TABLE 7 | Factors associated with physical activity among people with and without S-COVID-19-S. 9](#_Toc50492273)

[SUPPLEMEENTARY TABLE 8 | Factors associated with depression among people with and without S-COVID-19-S. 10](#_Toc50492274)

[SUPPLEMEENTARY TABLE 9 | Health literacy as a predictor associates with BMI among participants with and without suspected COVID-19 symptoms. 11](#_Toc50492275)

[SUPPLEMEENTARY TABLE 10 | Health literacy as a predictor associates with smoking status among participants with and without suspected COVID-19 symptoms. 12](#_Toc50492276)

[SUPPLEMEENTARY TABLE 11 | Health literacy as a predictor associates with drinking status among participants with and without suspected COVID-19 symptoms. 13](#_Toc50492277)

[SUPPLEMEENTARY TABLE 12 | Health literacy as a predictor associates with eating behavior among participants with and without suspected COVID-19 symptoms. 14](#_Toc50492278)

[SUPPLEMEENTARY TABLE 13 | Health literacy as a predictor associates with physical activity among participants with and without suspected COVID-19 symptoms. 15](#_Toc50492279)

[SUPPLEMEENTARY TABLE 14 | Health literacy as a predictor associates with depression among participants with and without suspected COVID-19 symptoms. 16](#_Toc50492280)

# **SUPPLEMEENTARY TABLE 1 |** Spearman's correlation coefficient of study variables for people without S-COVID-19-S.

|  | Age Group | Gender | Marital Status | Education | Occupation | CCI | Ability to pay medications | Social Status | BMI | Smoking Status | Drinking Status | Eating Behavior | Physical Activity | Depression | HL Index |
| --- | --- | --- | --- | --- | --- | --- | --- | --- | --- | --- | --- | --- | --- | --- | --- |
| Age Group | 1.00 |  |  |  |  |  |  |  |  |  |  |  |  |  |  |
| Gender | -0.12** | 1.00 |  |  |  |  |  |  |  |  |  |  |  |  |  |
| Marital Status | 0.02 | -0.07 | 1.00 |  |  |  |  |  |  |  |  |  |  |  |  |
| Education | -0.29** | 0.24** | 0.02 | 1.00 |  |  |  |  |  |  |  |  |  |  |  |
| Occupation | 0.14** | -0.13** | 0.02 | 0.08 | 1.00 |  |  |  |  |  |  |  |  |  |  |
| CCI | 0.11* | 0.004 | -0.02 | 0.06 | 0.09* | 1.00 |  |  |  |  |  |  |  |  |  |
| Ability to pay medications | -0.02 | 0.05 | 0.06 | 0.17** | 0.06 | -0.03 | 1.00 |  |  |  |  |  |  |  |  |
| Social Status | -0.18** | 0.07 | 0.15** | 0.36** | 0.04 | 0.01 | 0.18** | 1.00 |  |  |  |  |  |  |  |
| BMI | -0.05 | -0.01 | 0.004 | 0.05 | 0.04 | -0.01 | 0.05 | 0.10* | 1.00 |  |  |  |  |  |  |
| Smoking Status | -0.04 | 0.32** | 0.003 | 0.03 | -0.13** | -0.04 | 0.01 | -0.04 | 0.02 | 1.00 |  |  |  |  |  |
| Drinking Status | -0.12** | 0.42** | -0.04 | 0.17** | -0.11* | -0.03 | 0.06 | 0.01 | -0.06 | 0.34** | 1.00 |  |  |  |  |
| Eating Behavior | -0.04 | -0.05 | 0.05 | 0.24** | -0.03 | -0.06 | 0.02 | 0.11* | 0.05 | -0.01 | 0.003 | 1.00 |  |  |  |
| Physical Activity | -0.17** | 0.04 | -0.01 | 0.12** | 0.004 | -0.05 | 0.11* | 0.06 | 0.05 | 0.05 | 0.02 | 0.13** | 1.00 |  |  |
| Depression | 0.15** | -0.06 | -0.11* | -0.10* | -0.03 | 0.04 | -0.05 | -0.17** | -0.05 | 0.06 | 0.01 | -0.04 | -0.05 | 1.00 |  |
| HL Index | -0.29** | 0.15** | 0.10* | 0.44** | -0.02 | -0.10* | 0.18** | 0.31** | 0.08 | 0.06 | 0.06 | 0.17** | 0.10* | -0.08 | 1.00 |

*S-COVID-19-S, suspected COVID-19 symptoms; CCI, comorbidity; BMI, body mass index; HL, health literacy.*

**p<0.05; **p<0.01.*

# **SUPPLEMEENTARY TABLE 2 |** Spearman's correlation coefficient of study variables for people with S-COVID-19-S.

|  | Age Group | Gender | Marital Status | Education | Occupation | CCI | Ability to pay medications | Social Status | BMI | Smoking Status | Drinking Status | Eating Behavior | Physical Activity | Depression | HL Index |
| --- | --- | --- | --- | --- | --- | --- | --- | --- | --- | --- | --- | --- | --- | --- | --- |
| Age Group | 1.00 |  |  |  |  |  |  |  |  |  |  |  |  |  |  |
| Gender | 0.02 | 1.00 |  |  |  |  |  |  |  |  |  |  |  |  |  |
| Marital Status | 0.05 | 0.04 | 1.00 |  |  |  |  |  |  |  |  |  |  |  |  |
| Education | -0.20 | 0.09 | -0.05 | 1.00 |  |  |  |  |  |  |  |  |  |  |  |
| Occupation | 0.21** | -0.06 | 0.10* | -0.06 | 1.00 |  |  |  |  |  |  |  |  |  |  |
| CCI | 0.16** | -0.04 | 0.004 | -0.08 | 0.17** | 1.00 |  |  |  |  |  |  |  |  |  |
| Ability to pay medications | -0.01 | -0.05 | -0.11 | 0.10* | -0.02 | -0.04 | 1.00 |  |  |  |  |  |  |  |  |
| Social Status | -0.06 | 0.17** | 0.03 | 0.16** | 0.08 | 0.04 | 0.27** | 1.00 |  |  |  |  |  |  |  |
| BMI | -0.05 | 0.04 | 0.02 | 0.10* | 0.05 | 0.02 | -0.04 | 0.11* | 1.00 |  |  |  |  |  |  |
| Smoking Status | -0.03 | 0.22** | -0.01 | -0.05 | -0.09 | -0.02 | 0.02 | 0.04 | -0.05 | 1.00 |  |  |  |  |  |
| Drinking Status | -0.06 | 0.49** | 0.04 | 0.12* | -0.13 | 0.07 | -0.05 | 0.11* | 0.02 | 0.34** | 1.00 |  |  |  |  |
| Eating Behavior | -0.13 | 0.11* | -0.06 | 0.19** | 0.01 | -0.14 | -0.09 | 0.12* | 0.02 | -0.13 | -0.01 | 1.00 |  |  |  |
| Physical Activity | -0.22 | 0.001 | -0.06 | 0.25** | -0.06 | -0.14 | 0.07 | 0.01 | 0.03 | -0.13 | -0.05 | 0.32** | 1.00 |  |  |
| Depression | 0.14** | -0.09 | 0.04 | 0.01 | 0.08 | 0.001 | -0.09 | -0.20 | -0.08 | -0.01 | 0.01 | -0.20 | -0.04 | 1.00 |  |
| HL Index | -0.31** | 0.15** | -0.09 | 0.33** | -0.13 | -0.17 | 0.39** | 0.27** | 0.07 | 0.02 | 0.07 | 0.25** | 0.23** | -0.30 | 1.00 |

*S-COVID-19-S, suspected COVID-19 symptoms; CCI, comorbidity; BMI, body mass index; HL, health literacy.*

**p<0.05; **p<0.01.*

# **SUPPLEMEENTARY TABLE 3 |** Factors associated overweight/obesity among people with and without S-COVID-19-S.

| Variables | Without S-COVID-19-S | | With S-COVID-19-S | |
| --- | --- | --- | --- | --- |
|  | **OR (95% CI)^a^** | ***p*-value** | **OR (95% CI)^a^** | ***p*-value** |
| Age |  |  |  |  |
| 60 - 70 yrs. | 1.00 |  | 1.00 |  |
| 71 - 85 yrs. | 0.74 (0.42 - 1.29) | 0.285 | 0.70 (0.34 - 1.44) | 0.334 |
| Gender |  |  |  |  |
| Female | 1.00 |  | 1.00 |  |
| Male | 0.95 (0.59 - 1.53) | 0.841 | 1.34 (0.71 - 2.52) | 0.373 |
| Marital status |  |  |  |  |
| Never married | 1.00 |  | 1.00 |  |
| Ever married | 1.07 (0.30 - 3.77) | 0.921 | 1.47 (0.19 - 11.5) | 0.714 |
| Education |  |  |  |  |
| Elementary school or Illiterate | 1.00 |  | 1.00 |  |
| Junior high school | 0.94 (0.49 - 1.80) | 0.858 | 2.22 (0.67 - 7.29) | 0.189 |
| High school | 1.22 (0.61 - 2.45) | 0.582 | **5.09 (1.64 - 15.8)** | **0.005** |
| College/university or above | 1.44 (0.71 - 2.92) | 0.308 | 2.94 (0.92 - 9.42) | 0.069 |
| Occupation |  |  |  |  |
| Employed | 1.00 |  | 1.00 |  |
| Own business | 0.92 (0.24 - 3.55) | 0.903 | **0.16 (0.04 - 0.60)** | **0.007** |
| Others | 1.21 (0.34 - 4.27) | 0.768 | 0.35 (0.10 - 1.16) | 0.085 |
| Comorbidity |  |  |  |  |
| None | 1.00 |  | 1.00 |  |
| One or more | 0.96 (0.60 - 1.56) | 0.878 | 1.20 (0.60 - 2.40) | 0.601 |
| Ability to pay for medication |  |  |  |  |
| Very or fairly difficult | 1.00 |  | 1.00 |  |
| Very or fairly easy | 1.34 (0.83 - 2.17) | 0.232 | 0.71 (0.33 - 1.54) | 0.389 |
| Social status |  |  |  |  |
| Low | 1.00 |  | 1.00 |  |
| Middle or high | **2.38 (1.05 - 5.37)** | **0.038** | **2.90 (1.11 - 7.56)** | **0.030** |

*OR, odds ratio; CI, Confidence interval; S-COVID-19-S, suspected COVID-19 symptoms.*

*^a^ The unadjusted logistic regression model was used.*

# **SUPPLEMEENTARY TABLE 4 |** Factors associated with smoking behavior among people with and without S-COVID-19-S.

| Variables | Without S-COVID-19-S | | With S-COVID-19-S | |
| --- | --- | --- | --- | --- |
|  | **OR (95% CI)^a^** | ***p*-value** | **OR (95% CI)^a^** | ***p*-value** |
| Age, year |  |  |  |  |
| 60 - 70 | 1.00 |  | 1.00 |  |
| 71 - 85 | 0.74 (0.37 - 1.49) | 0.398 | 0.81 (0.44 - 1.50) | 0.499 |
| Gender |  |  |  |  |
| Women | 1.00 |  | 1.00 |  |
| Men | **14.43 (5.61 - 37.12)** | **<0.001** | **4.10 (2.19 - 7.68)** | **<0.001** |
| Marital status |  |  |  |  |
| Never married | 1.00 |  | 1.00 |  |
| Ever married | 0.93 (0.21 - 4.19) | 0.929 | 0.91 (0.20 - 4.14) | 0.902 |
| Education |  |  |  |  |
| Elementary school or Illiterate | 1.00 |  | 1.00 |  |
| Junior high school | 1.45 (0.65 - 3.23) | 0.370 | 0.60 (0.27 - 1.33) | 0.207 |
| High school | 1.50 (0.62 - 3.62) | 0.372 | 1.30 (0.62 - 2.71) | 0.491 |
| College/university or above | 1.27 (0.49 - 3.26) | 0.627 | 0.47 (0.20 - 1.12) | 0.090 |
| Occupation |  |  |  |  |
| Employed | 1.00 |  | 1.00 |  |
| Own business | 1.99 (0.42 - 9.38) | 0.382 | 1.26 (0.27 - 5.87) | 0.771 |
| Others | 0.75 (0.17 - 3.41) | 0.752 | 0.69 (0.15 - 3.23) | 0.635 |
| Comorbidity |  |  |  |  |
| None | 1.00 |  | 1.00 |  |
| One or more | 0.79 (0.43 - 1.45) | 0.439 | 0.88 (0.46 - 1.67) | 0.686 |
| Ability to pay for medication |  |  |  |  |
| Very or fairly difficult | 1.00 |  | 1.00 |  |
| Very or fairly easy | 1.05 (0.58 - 1.90) | 0.861 | 1.12 (0.60 - 2.09) | 0.719 |
| Social- status |  |  |  |  |
| Low | 1.00 |  | 1.00 |  |
| Middle or high | 0.75 (0.36 - 1.57) | 0.439 | 1.35 (0.68 - 2.65) | 0.391 |

*OR, odds ratio; CI, Confidence interval; S-COVID-19-S, suspected COVID-19 symptoms.*

*^a^ The unadjusted logistic regression model was used.*

# **SUPPLEMEENTARY TABLE 5 |** Factors associated with drinking behavior among people with and without S-COVID-19-S.

| Variables | Without S-COVID-19-S | | With S-COVID-19-S | |
| --- | --- | --- | --- | --- |
|  | **OR (95% CI)^a^** | ***p*-value** | **OR (95% CI)^a^** | ***p*-value** |
| Age, year |  |  |  |  |
| 60 - 70 | 1.00 |  | 1.00 |  |
| 71 - 85 | **0.50 (0.29 - 0.85)** | **0.010** | 0.71 (0.43 - 1.18) | 0.190 |
| Gender |  |  |  |  |
| Women | 1.00 |  | 1.00 |  |
| Men | **9.13 (5.42 - 15.4)** | **<0.001** | **20.54 (9.96 - 12.34)** | **<0.001** |
| Marital status |  |  |  |  |
| Never married | 1.00 |  | 1.00 |  |
| Ever married | 0.61 (0.22 - 1.66) | 0.331 | 1.79 (0.40 - 8.08) | 0.449 |
| Education |  |  |  |  |
| Elementary school or Illiterate | 1.00 |  | 1.00 |  |
| Junior high school | 1.36 (0.73 - 2.52) | 0.335 | 1.86 (0.90 - 3.83) | 0.093 |
| High school | 1.80 (0.93 - 3.49) | 0.082 | **2.63 (1.26 - 5.48)** | **0.010** |
| College/university or above | **3.31 (1.73 - 6.36)** | **<0.001** | **2.39 (1.17 - 4.89)** | **0.017** |
| Occupation |  |  |  |  |
| Employed | 1.00 |  | 1.00 |  |
| Own business | 0.42 (0.16 - 1.14) | 0.089 | 0.72 (0.23 - 2.21) | 0.565 |
| Others | **0.30 (0.12 - 0.76)** | **0.011** | 0.41 (0.13 - 1.24) | 0.114 |
| Comorbidity |  |  |  |  |
| None | 1.00 |  | 1.00 |  |
| One or more | 0.87 (0.56 - 1.34) | 0.520 | 1.43 (0.88 - 2.33) | 0.151 |
| Ability to pay for medication |  |  |  |  |
| Very or fairly difficult | 1.00 |  | 1.00 |  |
| Very or fairly easy | 1.35 (0.88 - 2.07) | 0.175 | 0.73 (0.43 - 1.25) | 0.254 |
| Social status |  |  |  |  |
| Low | 1.00 |  | 1.00 |  |
| Middle or high | 1.10 (0.61 - 1.97) | 0.759 | **2.00 (1.11 - 3.60)** | **0.020** |

*OR, odds ratio; CI, Confidence interval; S-COVID-19-S, suspected COVID-19 symptoms.*

*^a^ The unadjusted logistic regression model was used.*

# **SUPPLEMEENTARY TABLE 6 |** Factors associated with eating behavior among people with and without S-COVID-19-S.

| Variables | Without S-COVID-19-S | | With S-COVID-19-S | |
| --- | --- | --- | --- | --- |
|  | **OR (95% CI)^a^** | ***p*-value** | **OR (95% CI)^a^** | ***p*-value** |
| Age, year |  |  |  |  |
| 60 - 70 | 1.00 |  | 1.00 |  |
| 71 - 85 | 0.77 (0.43 - 1.39) | 0.386 | **0.46 (0.27 - 0.80)** | **0.006** |
| Gender |  |  |  |  |
| Women | 1.00 |  | 1.00 |  |
| Men | 0.77 (0.47 - 1.29) | 0.327 | **1.72 (1.08 - 2.72)** | **0.022** |
| Marital status |  |  |  |  |
| Never married | 1.00 |  | 1.00 |  |
| Ever married | 3.16 (0.41 - 24.14) | 0.267 | 0.51 (0.17 - 1.54) | 0.232 |
| Education |  |  |  |  |
| Elementary school or Illiterate | 1.00 |  | 1.00 |  |
| Junior high school | **2.75 (1.08 - 7.04)** | **0.035** | 1.25 (0.61 - 2.57) | 0.545 |
| High school | **4.35 (1.67 - 11.36)** | **0.003** | 0.94 (0.42 - 2.09) | 0.877 |
| College/university or above | **8.55 (3.35 - 21.85)** | **<0.001** | **3.57 (1.84 - 6.94)** | **<0.001** |
| Occupation |  |  |  |  |
| Employed | 1.00 |  | 1.00 |  |
| Own business | **0.32 (0.10 - 0.99)** | **0.047** | **0.32 (0.11 - 0.97)** | **0.043** |
| Others | **0.35 (0.13 - 0.97)** | **0.044** | 0.42 (0.14 - 1.22) | 0.110 |
| Comorbidity |  |  |  |  |
| None | 1.00 |  | 1.00 |  |
| One or more | 0.71 (0.42 - 1.19) | 0.191 | **0.40 (0.22 - 0.74)** | **0.004** |
| Ability to pay for medication |  |  |  |  |
| Very or fairly difficult | 1.00 |  | 1.00 |  |
| Very or fairly easy | 1.15 (0.70 - 1.90) | 0.587 | 0.60 (0.34 - 1.06) | 0.076 |
| Social status |  |  |  |  |
| Low | 1.00 |  | 1.00 |  |
| Middle or high | **2.96 (1.15 - 7.60)** | **0.024** | **2.14 (1.18 - 3.90)** | **0.013** |

*OR, odds ratio; CI, Confidence interval; S-COVID-19-S, suspected COVID-19 symptoms.*

*^a^ The unadjusted logistic regression model was used.*

# **SUPPLEMEENTARY TABLE 7 |** Factors associated with physical activity among people with and without S-COVID-19-S.

| Variables | Without S-COVID-19-S | | | | With S-COVID-19-S | | | |
| --- | --- | --- | --- | --- | --- | --- | --- | --- |
|  | **Physical activity-tertile-2^a^** | | **Physical activity-tertile-3^a^** | | **Physical activity-tertile-2^a^** | | **Physical activity-tertile-3^a^** | |
|  | **OR (95% CI)^b^** | ***p*-value** | **OR (95% CI)^b^** | ***p*-value** | **OR (95% CI)^b^** | ***p*-value** | **OR (95% CI)^b^** | ***p*-value** |
| Age, year |  |  |  |  |  |  |  |  |
| 60 - 70 | 1.00 |  | 1.00 |  | 1.00 |  | 1.00 |  |
| 71 - 85 | 1.12 (0.59 - 2.14) | 0.724 | **2.22 (1.44 - 3.44)** | **<0.001** | **2.01 (1.17 - 3.45)** | **0.011** | **2.99 (1.83 - 4.86)** | **<0.001** |
| Gender |  |  |  |  |  |  |  |  |
| Women | 1.00 |  | 1.00 |  | 1.00 |  | 1.00 |  |
| Men | 0.68 (0.37 - 1.25) | 0.215 | 0.84 (0.57 - 1.24) | 0.376 | 1.48 (0.88 - 2.47) | 0.138 | 0.94 (0.62 - 1.44) | 0.940 |
| Marital status |  |  |  |  |  |  |  |  |
| Never married | 1.00 |  | 1.00 |  | 1.00 |  | 1.00 |  |
| Ever married | 2.77 (0.72 - 10.7) | 0.140 | 1.30 (0.43 - 3.96) | 0.641 | 1.53 (0.36 - 6.54) | 0.567 | 2.22 (0.69 - 7.13) | 0.181 |
| Education |  |  |  |  |  |  |  |  |
| Elementary school or Illiterate | 1.00 |  | 1.00 |  | 1.00 |  | 1.00 |  |
| Junior high school | 0.65 (0.26 - 1.65) | 0.366 | **0.49 (0.27 - 0.89)** | **0.018** | **0.23 (0.11 - 0.48)** | **<0.001** | **0.22 (0.12 - 0.42)** | **<0.001** |
| High school | 1.11 (0.46 - 2.68) | 0.815 | 1.04 (0.59 - 1.84) | 0.884 | **0.37 (0.19 - 0.73)** | **0.004** | **0.29 (0.16 - 0.53)** | **<0.001** |
| College/university or above | 1.03 (0.38 - 2.78) | 0.95 | 1.13 (0.60 - 2.11) | 0.704 | **0.35 (0.17 - 0.76)** | **0.007** | 0.55 (0.30 - 1.01) | 0.055 |
| Occupation |  |  |  |  |  |  |  |  |
| Employed | 1.00 |  | 1.00 |  | 1.00 |  | 1.00 |  |
| Own business | 3.33 (0.80 - 13.9) | 0.100 | 1.98 (0.62 - 6.34) | 0.252 | 1.39 (0.25 - 7.85) | 0.708 | **3.74 (1.11 - 12.6)** | **0.033** |
| Others | 0.81 (0.37 - 1.76) | 0.597 | 0.89 (0.56 - 1.43) | 0.639 | 1.28 (0.77 - 2.12) | 0.345 | 1.10 (0.71 - 1.70) | 0.686 |
| Comorbidity |  |  |  |  |  |  |  |  |
| None | 1.00 |  | 1.00 |  | 1.00 |  | 1.00 |  |
| One or more | 0.76 (0.41 - 1.41) | 0.386 | 1.23 (0.83 - 1.82) | 0.305 | 1.60 (0.91 - 2.82) | 0.101 | **1.99 (1.21 - 3.26)** | **0.006** |
| Ability to pay for medication |  |  |  |  |  |  |  |  |
| Very or fairly difficult | 1.00 |  | 1.00 |  | 1.00 |  | 1.00 |  |
| Very or fairly easy | 1.18 (0.64 - 2.19) | 0.595 | **0.64 (0.43 - 0.94)** | **0.024** | 1.53 (0.82 - 2.84) | 0.184 | 0.65 (0.41 - 1.03) | 0.069 |
| Social status |  |  |  |  |  |  |  |  |
| Low | 1.00 |  | 1.00 |  | 1.00 |  | 1.00 |  |
| Middle or high | 0.90 (0.40 - 2.02) | 0.796 | 0.72 (0.43 - 1.22) | 0.220 | **1.82 (1.07 - 3.10)** | **0.026** | 0.87 (0.53 - 1.43) | 0.576 |

*OR, odds ratio; CI, Confidence interval; S-COVID-19-S, suspected COVID-19 symptoms.*

*^a^ Reference group is physical activity-tertile-1.*

*^b^The unadjusted multinomial logistic regression model was used.*

# **SUPPLEMEENTARY TABLE 8 |** Factors associated with depression among people with and without S-COVID-19-S.

| Variables | Without S-COVID-19-S | | With S-COVID-19-S | |
| --- | --- | --- | --- | --- |
|  | **OR (95% CI)^a^** | ***p*-value** | **OR (95% CI)^a^** | ***p*-value** |
| Age, year |  |  |  |  |
| 60 - 70 | 1.00 |  | 1.00 |  |
| 71 - 85 | **3.69 (1.58 - 8.64)** | **0.003** | **2.01 (1.28 - 3.17)** | **0.003** |
| Gender |  |  |  |  |
| Female | 1.00 |  | 1.00 |  |
| Male | 0.55 (0.22 - 1.36) | 0.193 | 0.65 (0.41 - 1.03) | 0.067 |
| Marital status |  |  |  |  |
| Never married | 1.00 |  | 1.00 |  |
| Ever married | **0.23 (0.06 - 0.85)** | **0.027** | 1.92 (0.43 - 8.68) | 0.393 |
| Education |  |  |  |  |
| Elementary school or Illiterate | 1.00 |  | 1.00 |  |
| Junior high school | **0.15 (0.04 - 0.55)** | **0.004** | **0.51 (0.27 - 0.96)** | **0.035** |
| High school | **0.25 (0.07 - 0.91)** | **0.035** | 0.55 (0.28 - 1.07) | 0.077 |
| College/university or above | 0.39 (0.12 - 1.24) | 0.111 | 1.01 (0.56 - 1.81) | 0.978 |
| Occupation |  |  |  |  |
| Employed | 1.00 |  | 1.00 |  |
| Own business | 1.13 (0.13 - 9.91) | 0.916 | 0.93 (0.25 - 3.48) | 0.916 |
| Others | 0.84 (0.11 - 6.71) | 0.871 | 1.37 (0.37 - 5.01) | 0.635 |
| Comorbidity |  |  |  |  |
| None | 1.00 |  | 1.00 |  |
| One or more | 1.44 (0.61 - 3.40) | 0.401 | 1.00 (0.61 - 1.65) | 0.991 |
| Ability to pay for medication |  |  |  |  |
| Very or fairly difficult | 1.00 |  | 1.00 |  |
| Very or fairly easy | 0.62 (0.27 - 1.45) | 0.272 | 0.60 (0.35 - 1.03) | 0.066 |
| Social status |  |  |  |  |
| Low | 1.00 |  | 1.00 |  |
| Middle or high | **0.22 (0.09 - 0.53)** | **0.001** | **0.36 (0.23 - 0.58)** | **<0.001** |

*OR, odds ratio; CI, Confidence interval; S-COVID-19-S, suspected COVID-19 symptoms.*

*^a^ The unadjusted logistic regression model was used.*

# **SUPPLEMEENTARY TABLE 9 |** Health literacy as a predictor associates with BMI among participants with and without suspected COVID-19 symptoms.

| Variables | Overweight/obese ( BMI ≥ 25.0 kg/m^2^) | |
| --- | --- | --- |
|  | **OR (95% CI)^a^** | ***p*-value** |
| Without S-COVID-19-S | | |
| HL index (1-score increment) | 1.01 (0.98 - 1.04) | 0.590 |
| Social status |  |  |
| Low | 1.00 |  |
| Middle or high | 2.22 (0.95 - 5.20) | 0.067 |
| With S-COVID-19-S | | |
| HL index (1-score increment) | 1.01 (0.96 - 1.06) | 0.806 |
| Education |  |  |
| Elementary school or illiterate | 1.00 |  |
| Junior high school | 2.51 (0.74 – 8.50) | 0.140 |
| High school | 5.42 (1.63 – 18.0) | 0.006 |
| College/university or above | 2.25 (0.64 – 7.92) | 0.206 |
| Occupation |  |  |
| Employed | 1.00 |  |
| Business owner | 0.18 (0.04 – 0.73) | 0.017 |
| Others | 0.42 (0.12 – 1.51) | 0.184 |
| Social status |  |  |
| Low | 1.00 |  |
| Middle or high | 2.27 (0.84 – 6.15) | 0.108 |

*BMI, body mass index; OR, odds ratio; CI, confidence interval; S-COVID-19-S, suspected COVID-19 symptoms; HL, health literacy.*

*^a^ The adjusted binary logistic regression model was used.*

# **SUPPLEMEENTARY TABLE 10 |** Health literacy as a predictor associates with smoking status among participants with and without suspected COVID-19 symptoms.

| Variables | Smoking | |
| --- | --- | --- |
|  | **OR (95% CI)^a^** | ***p*-value** |
| Without S-COVID-19-S | | |
| HL index (1-score increment) | 1.00 (0.96 - 1.04) | 0.975 |
| Gender |  |  |
| Women | 1.00 |  |
| Men | 14.4 (5.58 - 37.2) | <0.001 |
| With S-COVID-19-S | | |
| HL index (1-score increment) | 1.00 (0.96 - 1.04) | 0.948 |
| Gender |  |  |
| Women | 1.00 |  |
| Men | 4.12 (2.18 - 7.78) | <0.001 |

*OR, Odd ratio; CI, Confidence interval; S-COVID-19-S, suspected COVID-19 symptoms; HL, health literacy.*

*^a^ The adjusted binary logistic regression model was used.*

# **SUPPLEMEENTARY TABLE 11 |** Health literacy as a predictor associates with drinking status among participants with and without suspected COVID-19 symptoms.

| Variables | Drinking | |
| --- | --- | --- |
|  | **OR (95% CI)^a^** | ***p*-value** |
| Without S-COVID-19-S | | |
| HL index (1-score increment) | 0.99 (0.95 - 1.02) | 0.390 |
| Age, year |  |  |
| 60 - 70 | 1.00 |  |
| 71 - 85 | 0.62 (0.33 – 1.15) | 0.129 |
| Gender |  |  |
| Women | 1.00 |  |
| Men | 8.21 (4.78 – 14.1) | <0.001 |
| Education |  |  |
| Elementary school or illiterate | 1.00 |  |
| Junior high school | 0.90 (0.43 – 1.89) | 0.773 |
| High school | 1.14 (0.50 – 2.60) | 0.760 |
| College/university or above | 1.68 (0.70 – 4.02) | 0.245 |
| Occupation |  |  |
| Employed | 1.00 |  |
| Business owner | 0.56 (0.16 – 1.94) | 0.360 |
| Others | 0.46 (0.15 – 1.40) | 0.169 |
| With S-COVID-19-S | | |
| HL index (1-score increment) | 0.98 (0.94 - 1.03) | 0.378 |
| Gender |  |  |
| Women | 1.00 |  |
| Men | 20.1 (9.65 – 41.8) | <0.001 |
| Education |  |  |
| Elementary school or illiterate | 1.00 |  |
| Junior high school | 2.21 (0.97 – 5.03) | 0.059 |
| High school | 2.09 (0.90 – 4.86) | 0.085 |
| College/university or above | 2.48 (1.08 – 5.69) | 0.032 |
| Social status |  |  |
| Low | 1.00 |  |
| Middle or high | 1.40 (0.70 – 2.77) | 0.339 |

*OR, Odd ratio; CI, Confidence interval; S-COVID-19-S, suspected COVID-19 symptoms; HL, health literacy.*

*^a^ The adjusted binary logistic regression model was used.*

# **SUPPLEMEENTARY TABLE 12 |** Health literacy as a predictor associates with eating behavior among participants with and without suspected COVID-19 symptoms.

| Variables | Healthier diet | |
| --- | --- | --- |
|  | **OR (95% CI)^a^** | ***p*-value** |
| Without S-COVID-19-S | | |
| HL index (1-score increment) | 1.04 (0.99 - 1.08) | 0.103 |
| Education |  |  |
| Elementary school or illiterate | 1.00 |  |
| Junior high school | 2.08 (0.77 – 5.60) | 0.146 |
| High school | 3.11 (1.08 – 8.91) | 0.035 |
| College/university or above | 5.85 (2.01 – 17.0) | 0.001 |
| Occupation |  |  |
| Employed | 1.00 |  |
| Business owner | 0.87 (0.25 – 3.07) | 0.827 |
| Others | 0.64 (0.22 – 1.91) | 0.425 |
| Social status |  |  |
| Low | 1.00 |  |
| Middle or high | 1.41 (0.51 – 3.91) | 0.508 |
| With S-COVID-19-S | | |
| HL index (1-score increment) | 1.08 (1.04 - 1.13) | <0.001 |
| Age, year |  |  |
| 60 - 70 | 1.00 |  |
| 71 - 85 | 0.58 (0.31 – 1.10) | 0.094 |
| Gender |  |  |
| Women | 1.00 |  |
| Men | 1.65 (0.98 – 2.76) | 0.058 |
| Education |  |  |
| Elementary school or illiterate | 1.00 |  |
| Junior high school | 0.88 (0.40 – 1.94) | 0.755 |
| High school | 0.42 (0.17 – 1.01) | 0.053 |
| College/university or above | 1.96 (0.93 – 4.13) | 0.077 |
| Occupation |  |  |
| Employed | 1.00 |  |
| Business owner | 0.94 (0.26 – 3.45) | 0.928 |
| Others | 1.39 (0.93 – 4.13) | 0.608 |
| Comorbidity |  |  |
| None | 1.00 |  |
| One or more | 0.42 (0.22 – 0.83) | 0.012 |
| Social status |  |  |
| Low | 1.00 |  |
| Middle or high | 1.31 (0.68 – 2.55) | 0.424 |

*OR, Odd ratio; CI, Confidence interval; S-COVID-19-S, suspected COVID-19 symptoms; HL, health literacy.*

*^a^ The adjusted binary logistic regression model was used.*

# **SUPPLEMEENTARY TABLE 13 |** Health literacy as a predictor associates with physical activity among participants with and without suspected COVID-19 symptoms.

| Variables | Physical activity-tertile-2 | | Physical activity-tertile-3 | |
| --- | --- | --- | --- | --- |
|  | **OR (95% CI)^a^** | ***p*-value** | **OR (95% CI)^a^** | ***p*-value** |
| Without S-COVID-19-S |  |  |  |  |
| HL index (1-score increment) | 0.99 (0.95 - 1.03) | 0.639 | 0.99 (0.97 - 1.02) | 0.721 |
| Age, year |  |  |  |  |
| 60 - 70 | 1.00 |  | 1.00 |  |
| 71 - 85 | 1.02 (0.51 - 2.06) | 0.951 | 0.52 (0.32 - 0.83) | 0.007 |
| Education |  |  |  |  |
| Elementary school or illiterate | 1.00 |  | 1.00 |  |
| Junior high school | 1.74 (0.72 - 4.18) | 0.217 | 1.83 (1.04 - 3.21) | 0.036 |
| High school | 1.81 (0.65 - 5.05) | 0.255 | 1.82 (0.95 - 3.47) | 0.071 |
| College/university or above | 1.82 (0.62 - 5.28) | 0.274 | 1.57 (0.79 - 3.09) | 0.196 |
| Ability to pay for medications |  |  |  |  |
| Very or fairly difficult | 1.00 |  | 1.00 |  |
| Very or fairly easy | 0.82 (0.44 - 1.56) | 0.552 | 1.54 (1.02 - 2.31) | 0.04 |
| With S-COVID-19-S |  |  |  |  |
| HL index (1-score increment) | 1.00 (0.96 - 1.04) | 0.952 | 1.04 (1.01 - 1.08) | 0.023 |
| Age, year |  |  |  |  |
| 60 - 70 | 1.00 |  | 1.00 |  |
| 71 - 85 | 0.54 (0.29 - 0.99) | 0.045 | 0.44 (0.25 - 0.75) | 0.003 |
| Education |  |  |  |  |
| Elementary school or illiterate | 1.00 |  | 1.00 |  |
| Junior high school | 1.34 (0.62 - 2.90) | 0.457 | 0.89 (0.45 - 1.75) | 0.727 |
| High school | 1.37 (0.57 - 3.30) | 0.485 | 1.45 (0.72 - 2.94) | 0.297 |
| College/university or above | 4.70 (2.09 - 10.59) | <0.001 | 2.98 (1.47 - 6.04) | 0.002 |
| Social Status |  |  |  |  |
| Low | 1.00 |  | 1.00 |  |
| Middle or high | 1.51 (0.24 - 9.39) | 0.662 | 0.61 (0.16 - 2.34) | 0.469 |
| Comorbidity |  |  |  |  |
| None | 1.00 |  | 1.00 |  |
| One or more | 1.42 (0.23 - 8.70) | 0.703 | 0.68 (0.18 - 2.55) | 0.566 |
| Occupation |  |  |  |  |
| Employed | 1.00 |  | 1.00 |  |
| Business owner | 0.69 (0.38 - 1.26) | 0.228 | 0.62 (0.36 - 1.06) | 0.083 |
| Others | 0.46 (0.25 - 0.82) | 0.009 | 0.75 (0.43 - 1.31) | 0.312 |

*OR, Odd ratio; CI, Confidence interval; S-COVID-19-S, suspected COVID-19 symptoms; HL, health literacy.*

*^a^ The adjusted multinomial logistic regression model was used.*

# **SUPPLEMEENTARY TABLE 14 |** Health literacy as a predictor associates with depression among participants with and without suspected COVID-19 symptoms.

| Variables | Depression (PHQ ≥ 10) | |
| --- | --- | --- |
|  | **OR (95% CI)^a^** | ***p*-value** |
| Without S-COVID-19-S |  |  |
| HL index (1-score increment) | 1.02 (0.96 - 1.09) | 0.461 |
| Age, year |  |  |
| 60 - 70 | 1.00 |  |
| 71 - 85 | 2.75 (1.05 – 7.17) | 0.039 |
| Marital status |  |  |
| Never married | 1.00 |  |
| Ever married | 0.33 (0.08 – 1.45) | 0.143 |
| Education |  |  |
| Elementary school or illiterate | 1.00 |  |
| Junior high school | 0.26 (0.07 – 1.06) | 0.060 |
| High school | 0.51 (0.11 – 2.29) | 0.378 |
| College/university or above | 0.66 (0.16 – 2.79) | 0.571 |
| Social status |  |  |
| Low | 1.00 |  |
| Middle or high | 0.33 (0.12 – 0.94) | 0.037 |
| With S-COVID-19-S | | |
| HL index (1-score increment) | 0.91 (0.87 - 0.94) | <0.001 |
| Age, year |  |  |
| 60 - 70 | 1.00 |  |
| 71 - 85 | 1.30 (0.76 – 2.20) | 0.335 |
| Education |  |  |
| Elementary school or illiterate | 1.00 |  |
| Junior high school | 0.84 (0.41 – 1.72) | 0.637 |
| High school | 1.40 (0.64 – 3.07) | 0.402 |
| College/university or above | 2.73 (1.35 – 5.52) | 0.005 |
| Social status |  |  |
| Low | 1.00 |  |
| Middle or high | 0.42 (0.25 – 0.71) | 0.001 |

*PHQ, patient health questionnaire; OR, Odd ratio; CI, Confidence interval; S-COVID-19-S, suspected COVID-19 symptoms; HL, health literacy.*

*^a^ The adjusted binary logistic regression model was used.*
